# The Pencil Fulcrum Technique: A Simple Method for Closed Reduction of Extra-Octave Fractures in Children

**DOI:** 10.1055/s-0045-1813231

**Published:** 2025-12-15

**Authors:** Vivek M. Sodhai, Darshan Munot, Rahul Jaiswal, Sandeep Patwardhan

**Affiliations:** 1 Department of Pediatric Orthopedics, Sancheti Institute for Orthopaedics & Rehabilitation, Pune, India

**Keywords:** closed fracture reduction, finger phalanges, fractures, bone, pediatrics, falanges dos dedos da mão, fraturas ósseas, pediatria, redução fechada

## Abstract

Extra-octave fractures of the fifth proximal phalanx are Salter-Harris type II physeal injuries resulting in ulnar deviation and dorsal angulation. If inadequately reduced, these fractures may result in cosmetic deformity, malalignment, and functional impairment. Traditional reduction methods, such as the Jahss maneuver, can be technically demanding, forceful, and often require sedation or surgical assistance, which limits their use in younger children and in resource-constrained environments.

We report our experience with the fulcrum-assisted “pencil technique” for closed reduction in four children, aged 4 to 11 years, under digital nerve block without sedation or traction. The method employs a simple pencil placed at the base of the fourth web space near the metacarpophalangeal joint, serving as a fulcrum. Gentle adduction and flexion pressure is applied to the proximal phalanx, correcting both ulnar deviation and dorsal angulation by biomechanical leverage. Reduction was confirmed clinically and radiographically, and immobilization was achieved with buddy strapping or tin splinting for 3 weeks.

All patients achieved stable reduction and demonstrated uneventful healing, pain-free full range of motion, and return to baseline activities at follow-up ranging from 6 to 12 weeks. Functional outcomes assessed by the quick disabilities of the arm, shoulder, and hand (QuickDASH) scores were excellent: two patients scored 0, one 2.27, and another 4.55. No complications or redisplacements were observed.

The pencil fulcrum technique is simple, safe, reproducible, and cost-effective. It represents a valuable addition to the armamentarium of physicians managing pediatric extra-octave fractures, particularly in outpatient, emergency, and low-resource settings.

## Resumo

As fraturas extra-oitavas da quinta falange proximal são lesões fisárias do tipo II de Salter-Harris e provocam desvio ulnar e angulação dorsal. A redução inadequada dessas fraturas pode causar deformidade estética, perda de alinhamento e comprometimento funcional. Métodos tradicionais de redução, como a manobra de Jahss, podem ser tecnicamente complexas, agressiva e frequentemente exigem sedação ou cirurgia, o que limita seu uso em crianças pequenas e ambientes com recursos limitados.

Relatamos nossa experiência com a “técnica do lápis” assistida por fulcro para redução fechada em quatro crianças, com idades entre 4 e 11 anos, sob bloqueio nervoso digital sem sedação ou tração. O método emprega um lápis simples, que atua como fulcro, posicionado na base do quarto espaço interdigital próximo à articulação metacarpofalangiana. Uma leve pressão de adução e flexão é aplicada à falange proximal, o que corrige o desvio ulnar e a angulação dorsal por alavancagem biomecânica. A redução foi confirmada clínica e radiograficamente, realizando-se imobilização solidária com a técnica de “buddy strapping” ou tala de estanho, mantida por 3 semanas.

Na consulta de acompanhamento, realizada 6 a 12 semanas após o procedimento, todos os pacientes apresentaram redução estável, cicatrização sem intercorrências, amplitude de movimento completa e indolor, e retorno às atividades básicas. Os resultados funcionais avaliados pelo escore Quick Disabilities of the Arm, Shoulder and Hand (QuickDASH) foram excelentes: dois pacientes com pontuação 0, um com 2,27 e o último com 4,55. Não foram observadas complicações ou novas luxações.

A técnica do fulcro com lápis é simples, segura, reprodutível e econômica. Representa uma valiosa adição ao arsenal de médicos para tratamento de fraturas extra-oitavas pediátricas, particularmente em ambulatórios, emergências e instituições com poucos recursos.

## Introduction

Pediatric hand fractures are among the most common injuries seen in emergency and orthopedic settings, often resulting from sports, falls, or direct trauma. Within this spectrum, fractures involving the base and neck of the fifth proximal phalanx are frequently encountered, particularly due to axial loading or impact to the ulnar border of the hand. One specific variant, known as the “extra-octave fracture”, is a Salter-Harris type II injury of the fifth proximal phalanx's base. 1

**Fig. 1:**
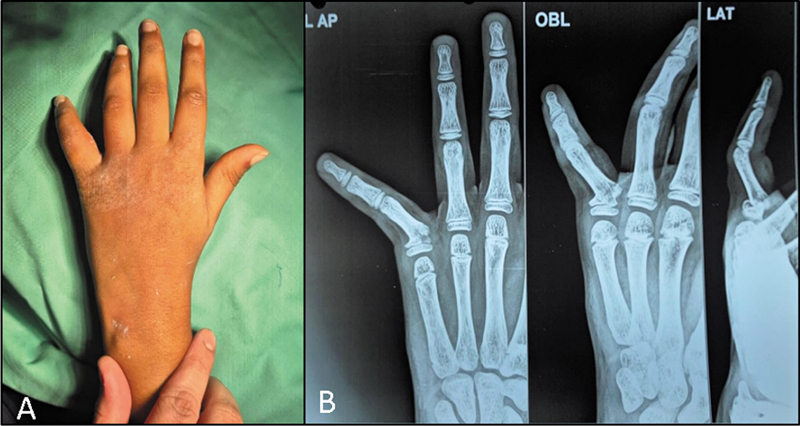
( **A** ) Clinical image showing the ulnar deviation and dorsal angulation in a “extra-octave fracture”. ( **B** ) Radiograph showing Salter-Harris type II injury on the fifth proximal phalanx.

The term “extra-octave fracture” was given to the increased ulnar deviation and dorsal angulation of the little finger that allows a theoretical extension of hand span similar to a pianist reaching an extra octave, at the cost of function and alignment ( Fig. 1 ). 1 2

Historically, these injuries were often misclassified or grouped with other juxtaepiphyseal fractures until the unique angulation and anatomical implications of the extra-octave variant were better recognized. Early literature noted that, although these fractures might appear innocuous, improper reduction could lead to cosmetic deformity, rotational malalignment, and loss of fine motor control, particularly in tasks requiring a strong ulnar grip. 1 2

Conventional management of these fractures involves closed reduction using traction-based methods such as the Jahss maneuver (90-90 flexion-pressure technique), which was first described for metacarpal neck fractures, and later adapted for phalangeal base injuries, including extra-octave ones. 3 This maneuver involves flexing the metacarpophalangeal joint (MCPJ) and applying force in a volar direction on the metacarpal shaft and a dorsal direction on the proximal interphalangeal (PIP) joint, along with immobilization to ensure the finger is not in extension. Forceful reduction with this technique can worsen the soft tissue injury. Al-Qattan recommends immobilization with an ulnar gutter splint or cast with the MCPJ flexed at 90°. 1

Given these challenges, there is an increasing shift towards simple, reproducible, and less invasive techniques that can be safely performed in outpatient settings or emergency departments. In this context, we used a novel fulcrum-assisted method described as “the pencil method,” which was introduced by Beatty et al. 4 It employs a standard pencil or a similar solid cylindrical object to achieve closed reduction through biomechanical leverage, reaching anatomical reduction of these fractures. 4

Hereby, we present a series of four children with extra-octave fractures treated with fulcrum-based reduction under digital block, using a pencil or a similar solid cylindrical object without any surgical tools.

## Methods

The Institutional Review Board (IRB) approved this retrospective study and an informed consent was taken from the guardians of all patients for data and image representation. We conducted a retrospective case series of 4 pediatric patients, aged 4 to 11 years, who presented with extra-octave fractures of the 5 ^th^ proximal phalanx, between 2023 and 2024.

All fractures were classified as Salter–Harris type II at the base of the proximal phalanx. Patients were treated in the outpatient clinic or emergency department using a fulcrum-assisted closed reduction technique, performed under digital nerve block with 2% lidocaine. All patients were assessed for radiological healing and the Quick Disabilities of the Arm, Shoulder, and Hand (QuickDASH) questionnaire for functional recovery.

## Technique Description

**Fig. 2:**
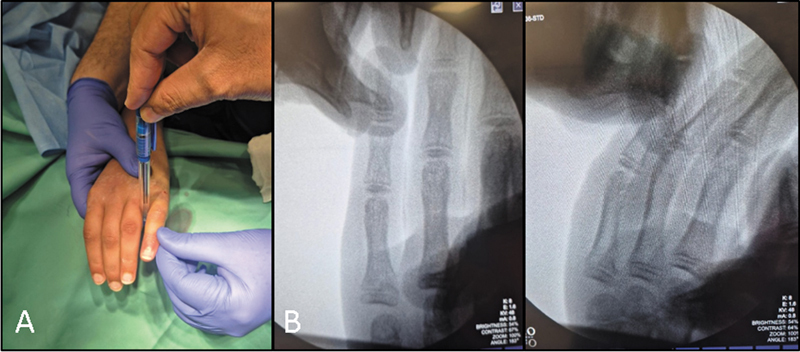
( **A** ) Demonstration of the fulcrum-assisted reduction with pencil in the fourth web space near the MCPJ. ( **B** ) Intraoperative fluoroscopy image showing anatomical reduction.

**Fig. 3:**
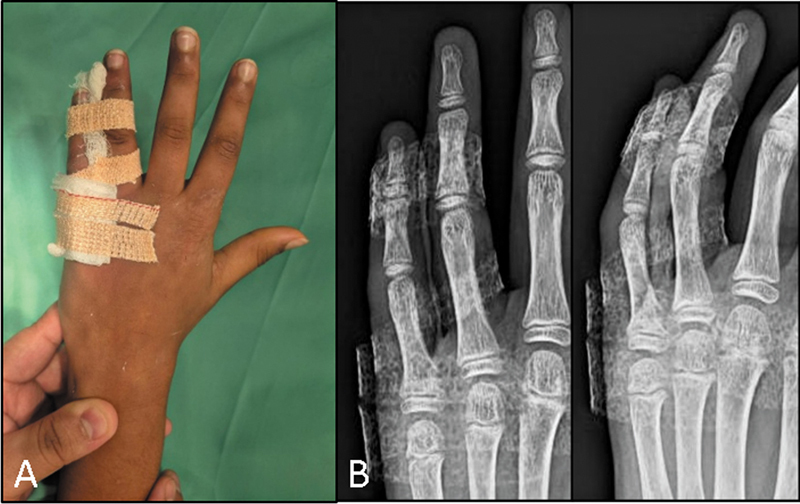
( **A** ) Postoperative immobilization with buddy strapping. ( **B** ) Postoperative radiograph showing a stable fracture reduction.

1. Position the patient supine with the affected hand pronated on a hand table.
2. Administer a digital nerve block using 2% lidocaine to the finger involved.
3. Clinical deformity of the fifth digit and the fracture was visualized.
4. Place a pencil or a similar solid cylindrical object perpendicular to the table at the base of the fourth web space, near the MCPJ ( Fig. 2A ).
5. Using the pencil as a fulcrum, gentle pressure was applied to adduct and flex the proximal phalanx. This coordinated movement facilitated fracture reduction through biomechanical leverage, correcting both the ulnar deviation and the dorsal angulation.
6. The reduction was confirmed clinically and radiographically using fluoroscopy in anteroposterior, oblique, and lateral views ( Fig. 2B ).
7. The finger was immobilized using buddy strapping with or without a tin splint for a duration of 3 weeks ( Fig. 3A ). Radiographic imaging was taken in the immediate postoperative period ( Fig. 3B ).
8. Range-of-motion exercises were initiated at the end of 3 weeks, followed by grip-strengthening exercises.

## Results

All fractures in our cohort occurred in the dominant right hand. All four patients underwent successful closed reduction with the pencil fulcrum technique without the need for traction, sedation, or surgical instrumentation. Radiographic assessment confirmed anatomical alignment in each case, which was maintained until bone union. Immobilization was well tolerated.

Fracture healing was achieved uneventfully in all cases, with no instances of redisplacement, infection, or neurovascular compromise. At final follow-up, which ranged from 3 to 6 months, every patient demonstrated full, pain-free range of motion of the affected finger and returned to preinjury levels of activity. Functional outcomes assessed using the QuickDASH questionnaire showed excellent recovery, with scores of 0 in 2 cases, 2.27 in one, and 4.55 in another ( Table 1 ).

**Table 1:** Patient characteristics, injury details, follow-up, and functional outcomes (QuickDASH)

Patient	Age, years	Injured hand	Dominant hand	Mechanism	Type of injury	Follow-up	QuickDASH score
**1**	4	Left	Right	Fell on an outstretched hand	Salter-Harris II – base of proximal phalanx	3 months	4.55
**2**	11	Left	Right	Direct trauma	Salter-Harris II – base of proximal phalanx	6 months	0
**3**	9	Left	Right	Injury while catching a cricket ball	Salter-Harris II – base of proximal phalanx	4 months	2.27
**4**	10	Left	Right	Direct trauma by a football	Salter-Harris II – base of proximal phalanx	6 months	0

**Abbreviations:** QuickDASH, quick Disability of the arm, shoulder, and hand questionnaire).

## Discussion

Although relatively uncommon, extra-octave fractures represent a distinct subset of pediatric proximal phalangeal injuries that demand careful management to prevent long-term deformity and dysfunction. The characteristic ulnar deviation and dorsal angulation can result in significant functional impairment if inadequately reduced, especially in activities requiring strong ulnar grip and fine motor control. 1 2 Traditional closed reduction methods, such as the Jahss maneuver, 3 have been adapted for these injuries but remain technically challenging and uncomfortable for children.

The Jahss maneuver, originally described for metacarpal neck fractures, involves 90° flexion of the MCP joint and opposing dorsal-volar pressure across the fracture site. While effective in experienced hands, the technique is inherently forceful, often painful, and frequently necessitates sedation or even general anesthesia in younger patients. Furthermore, reduction attempts using traction-based maneuvers can exacerbate soft tissue injury and may have a predisposition to redisplacement. These interventions also carry procedural risks and increase healthcare utilization, including potential operating room time and resource allocation. There has been a growing emphasis on developing alternative reduction strategies that are both minimally invasive and feasible even in remote nursing homes or clinics.

In contrast, the pencil fulcrum technique offers a biomechanically elegant and patient-friendly alternative. This technique was originally described by Beatty et al. 4 By using a simple cylindrical object, such as a pencil, as a fulcrum at the base of the fourth web space, the surgeon can apply gentle adduction and flexion forces that leverage the proximal phalanx into anatomical alignment. This technique minimizes traction and avoids excessive manipulation, thereby reducing discomfort and eliminating the need for sedation in most cases. Our results demonstrated that the maneuver is both effective and well tolerated under digital block alone.

Other reduction methods, such as percutaneous pin-assisted reduction or mini-open approaches, are reserved for irreducible or unstable patterns. While these techniques provide reliable outcomes, they increase procedural complexity and resource utilization. They also carry risks of infection, physeal injury, and anesthesia-related complications. In contrast, the pencil fulcrum method requires no specialized equipment, can be performed rapidly in outpatient or emergency settings, and avoids the morbidity associated with operative intervention.

Our small series supports the reproducibility and safety of this maneuver. All patients achieved stable reductions with no complications, and functional outcomes were excellent at follow-up. It is important to highlight that the absence of redisplacement underscores the biomechanical soundness of this fulcrum-assisted approach when applied to stable fracture patterns.

Limitations of our study include the small cohort size, short-term follow-up, and lack of a comparative control group. The shorter follow-up can be attributed to a shorter recovery time in these children. Furthermore, this technique is not appropriate for comminuted, open, grossly unstable fractures, or cases with soft tissue interposition. Its applicability in older adolescents or adults, with stiffer bones and soft tissues, remains to be determined.

## Final Considerations

The fulcrum-assisted pencil technique is a simple, safe, reproducible and cost-effective method for closed reduction of extra-octave fractures in children. Its minimal reliance on specialized tools or sedation makes it an excellent option for outpatient clinics, emergency departments, and resource-limited setups. Given its reproducibility and cost-effectiveness, this technique offers a valuable addition to the orthopedic clinician's armamentarium. Future studies involving larger, multi-center cohorts and longer-term follow-up are warranted to further validate its efficacy and optimize clinical guidelines for management.

## Data Availability

Data will be available upon request to the corresponding author.

## References

[1] Szymanski S Zylstra M Hull A “One Note Higher”: A Unique Pediatric Hand Fracture Clin Pract Cases Emerg Med 2021 5 02 270 272Doi: 10.5811/cpcem.2021.3.51806 34437026 10.5811/cpcem.2021.3.51806 PMC8143814

[2] Mims L Khodaee M Extra-Octave Fracture in a 14-Year-Old Basketball Player J Pediatr 2017 186 206 2060 10.1016/j.jpeds.2017.03.018 28408129

[3] Jahss S A Fractures of the metacarpals: a new method of reduction and immobilization J Bone Joint Surg Am 1938 20 01 178 186

[4] Beatty E Light T R Belsole R J Ogden J A Wrist and hand skeletal injuries in children Hand Clin 1990 6 04 723 738Doi: 10.1016/S0749-0712(21)01068-4 2269682

